# Protein-induced membrane asymmetry modulates OMP folding kinetics and stability†

**DOI:** 10.1039/d4fd00180j

**Published:** 2025-01-10

**Authors:** Jonathan M. Machin, Neil A. Ranson, Sheena E. Radford

**Affiliations:** a Astbury Centre for Structural Molecular Biology, School of Molecular and Cellular Biology, Faculty of Biological Sciences, University of Leeds Leeds LS2 9JT UK j.m.machin@leeds.ac.uk s.e.radford@leeds.ac.uk

## Abstract

Biological membranes are asymmetric structures, with asymmetry arising from differences in lipid identity in each leaflet of the bilayer, as well as non-uniform distribution of lipids and small molecules in the membrane. Proteins can also induce and modulate membrane asymmetry based on their shape, sequence and interactions with lipids. How membrane asymmetry affects macromolecular behaviour is poorly understood because of the complexity of natural membrane systems, and difficulties in creating relevant asymmetric bilayer systems *in vitro*. Here, we present a method exploiting the efficient, unidirectional folding of the transmembrane β-barrel outer membrane protein, OmpA, to create asymmetric proteoliposomes with protein-induced dipoles of known direction (arising from sequence variation engineered into the OmpA loops). We then characterise the folding kinetics and stability of different OmpA variants into these proteoliposomes. We find that both the primary sequence of the folding OmpA and the dipole of the membrane into which folding occurs play an important role for modulating the rate of folding. Critically, we find that by complementarily matching the charge on the folding protein to the membrane dipole it is possible to enhance both the folding kinetics and the stability of the folded OmpA. The results hint at how cells might exploit loop charge in membrane-embedded proteins to manipulate membrane environments for adaptation and survival.

## Abstract 2

Complementary charge matching between a protein-induced membrane dipole and a folding OMP leads to optimal folding kinetics and protein stability.

## Graphical abstract

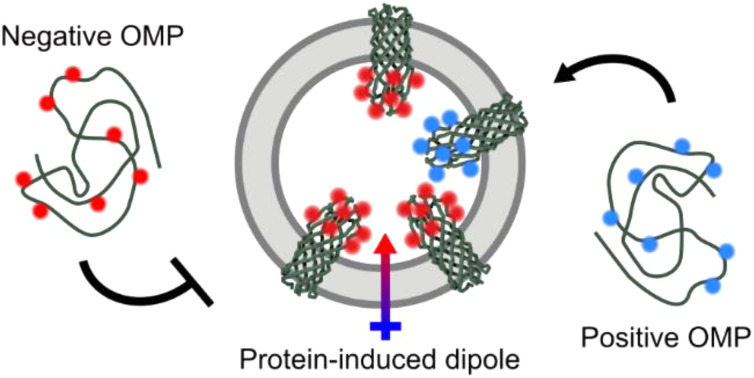

## Introduction

Biological membranes consist of lipid bilayers associated with a diverse set of other intra-membrane and membrane-associated entities, and are essential for many cellular processes, including compartmentalisation, signalling, transport and cellular protection.^1,2^ Membrane asymmetry has been implicated in an array of essential biological processes including apoptosis,^3^ cell morphology,^4^ protein–lipid interactions^5,6^ and modulating enzyme activity.^7^*In vivo*, membrane asymmetry arises from multiple sources^8,9^ including lipid acyl chain and lipid headgroup bilayer leaflet asymmetry, polarised organisation of hydrophobic compounds dissolved in the membrane, and asymmetric differences induced by the presence of transmembrane and peripherally attached proteins at the membrane surface.^10^

While the effects of lipid asymmetry on protein folding and stability have been reported recently using bacterial outer membrane proteins (OMPs) as a model system,^11^ and the effects of lipid asymmetry on protein insertion into the membrane has been explored for other proteins,^12,13^ the implications of protein-induced membrane asymmetry on protein folding and stability remains poorly understood. Transmembrane proteins confer asymmetry to the membrane *via* differences in the residues they expose on their membrane-facing surfaces,^14,15^ as well as by their shape and structural properties.^16^ Asymmetry in natural membranes hence arises *via* multiple mechanisms, including local enrichment of different lipids in the bilayer leaflets,^15,17^ tension and curvature in the bilayer that is induced or enhanced by proteins and manifested unevenly across the bilayer,^18,19^ and alteration in the electrostatic potential of embedded proteins that produces local dipoles, that may work in concert with lipid-induced charge asymmetry.^20–23^ Often these features are combined, for example, piezo ion channels induce membrane asymmetry by altering the relative curvature of each side of the bilayer, locally enriching lipids in different leaflets of the bilayer and acting in concert to manipulate global membrane disorder.^17,19,24,25^ While individual proteins asymmetrically modulate their local membrane context, long-range effects can also emerge *via* reinforcement across multiple proteins,^10,19,23^ especially in protein-rich membranes or protein arrays. Indeed, most membranes contain a high concentration of proteins in their bilayers. For example, the inner membrane of diderm bacteria has a lipid : protein ratio (LPR) of ∼32 : 1 (mol mol^−1^), with proteins covering about 25% of the membrane's surface area.^26^

**Fig. 1:**
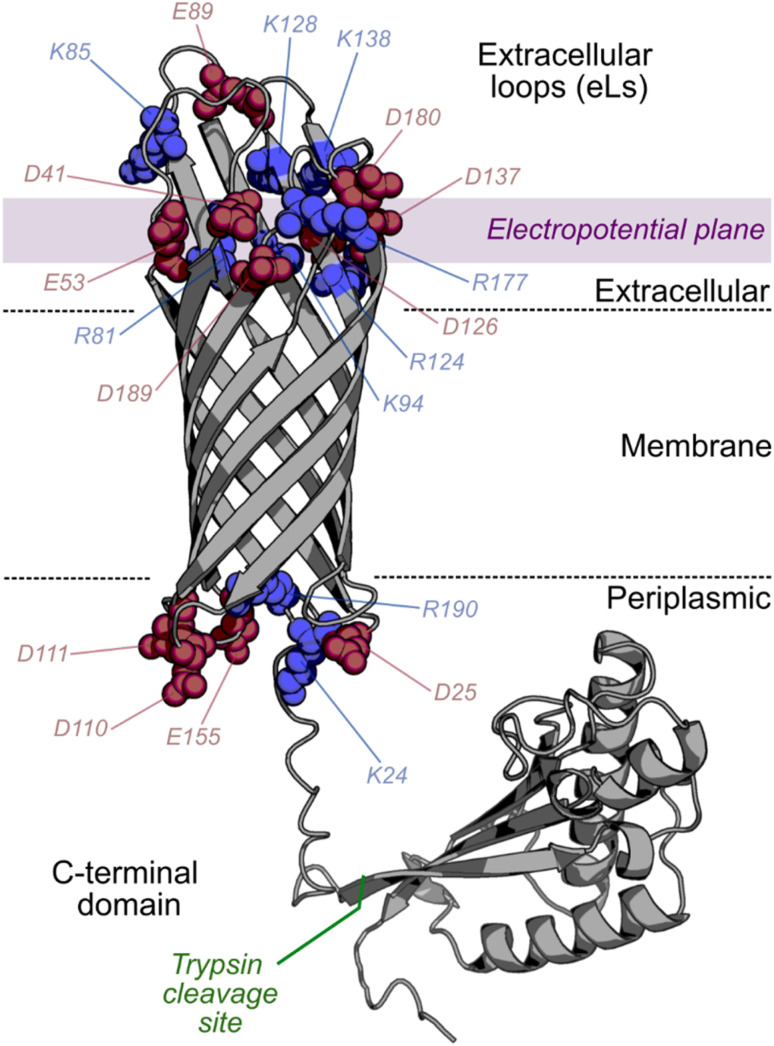
Structural overview and charged residues of OmpA. Full-length OmpA has a transmembrane β-barrel and a natively periplasmic soluble domain joined by a flexible linker. All solvent-accessible charged residues in the OmpA barrel (following trypsin cleavage, site marked in green) are shown as spheres and labelled (red: negative, blue: positive). The electropotential plane indicates the approximate region used to calculate the electrostatic potential shown in Fig. 2a. OmpA-Neg neutralises all labelled positive extracellular residues (R81S, K85T, K94S, R124S, K128G, K134S and R177S). OmpA-Pos neutralises all labelled negative extracellular residues (D41S, E53N, E89V, D126S, D137S, D180S and D189S). OmpA-Neut neutralises all extracellular positive and negative residues by combining the OmpA-Pos/-Neg mutations. (OmpA modelled from PDBs 1G90 (ref. 1) (transmembrane) and 2MQE^2^ (C-terminal domain)).

The outer membrane (OM) of diderm bacteria is a highly unusual and grossly asymmetric membrane. OMPs embedded in the OM exhibit low, and highly restricted, diffusion,^27^ in part because of the extremely low LPR in the OM (∼7 : 1 (ref. 26 and 28)). In addition, the outer leaflet of the OM is dominated by lipopolysaccharides, with phospholipids in the inner leaflet, making the OM one of the most profoundly asymmetric membranes in biology.^29^ The dense packing of OMPs in the OM also increases the likelihood of potential effects of protein-induced membrane asymmetry on a local or long-range scale *in vivo*. OMPs are highly stable β-barrels 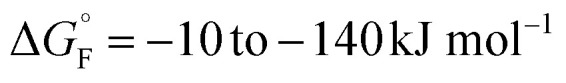,^28^ with transmembrane β-strands typically linked by long (>8-residue) extracellular loops and short (<5-residue) intracellular turns.^30^ OmpA is a well-studied OMP,^28,31^ that is common in the OM (>100 000 copies per cell^32^ in *Escherichia coli* (*E. coli*)) and confers strength and resistance (*e.g.* resilience to enhanced osmotic pressure^33^) to the cell. Natively folded OmpA consists of an eight-stranded transmembrane barrel domain linked by four extracellular loops (13–18 residues in length) and three short (4-residue) turns in the periplasmic face^34,35^ (Fig. 1). It also possesses an ∼15 kDa C-terminal (natively intracellular (periplasmic)) soluble domain that readily refolds *in vitro*^36,37^ (Fig. 1). Altering membrane properties has been shown to modulate the folding of OMPs (including OmpA), for example, changing lipid acyl chain length^38^ and/or head group identity,^39^ or altering the global membrane properties such as lipid order,^40^ and the presence of membrane defects.^41^ While decreasing LPR generally reduces the folding rate of OMPs,^28^ the effect of LPR on folding rate and yield of OmpA in different lipids and lipid mixtures has not been studied systematically to date.

The charge on proteins and membranes is known to affect protein folding, localisation and function,^42–44^ including the ‘positive-inside’ rule for transmembrane helix topology determination^45,46^ and ‘positive-outside’ rule for OMPs.^47,48^ We have recently shown that non-uniform charge distribution across a lipid bilayer, generated by asymmetric lipid content between the bilayer leaflets, can modulate the folding and stability of OMPs.^11^ Here, using OmpA as a platform, we have generated proteoliposomes with different protein-induced bilayer charge dipoles of known orientation and used them to determine the effect of protein-induced membrane dipoles on the folding rates and stability of OmpA variants. We show that protein-induced charge asymmetries indeed modulate the folding rates of the different OmpA variants and, most importantly, demonstrate that it is possible to enhance the folding rate and stability of OmpA by complementarily matching its extracellular loop charge to that of the protein-induced membrane dipole it is folding into. The results have implications on how bilayer asymmetry can alter OMP folding. In addition, they present a robust method for exploring the effects of protein-mediated bilayer asymmetry on membrane protein behaviour more broadly, and inform principles for the design and generation of biosynthetic membranes containing OMPs as pores, channels or sensors for translational applications.^49–51^

## Methods

### Electrostatic modelling

To model the electrostatic environment for the OmpA variant of interest, the protein (modelled from PDBs 1G90 (ref. 52) (transmembrane) and 2MQE^53^ (C-terminal domain)) was placed in an all-atom membrane of 1,2-dilauroyl-*sn*-glycero-3-phosphocholine (DLPC) (20 × 20 × 14 nm box) with explicit water neutralised with 50 mM NaCl (set-up using CHARMM-GUI^54,55^) and equilibrated for 10 ns using Gromacs.^56^ The final simulation frame was processed through APBS^57^ and the resulting electrostatic potentials were analysed and figures drawn using custom python scripts, analysing a slice of about 0.6 nm thickness parallel to the membrane plane centred on the protein.

### OmpA purification

OmpA and its variants were expressed in *E. coli* BL21(DE3) as inclusion bodies and purified in an unfolded state as described previously.^11^ The OmpA variants created were: OmpA-Neg: R81S, K85T, K94S, R124S, K128G, K134S and R177S; OmpA-Pos: D41S, E53N, E89V, D126S, D137S, D180S and D189S; OmpA-Neut: containing the combined mutations from OmpA-Pos and OmpA-Neg.^11^ Mutants were chosen by identifying the most common naturally occurring sequence variant at each position or, if not conserved, serine was used.

### Liposome preparation and initial folding

The required amount of resuspended lipid (DLPC, or dimyristoylphosphatidylcholine (DMPC) (Avanti polar lipids) in 1 : 4 MeOH : chloroform) was dried to a thin film in a glass vial and desiccated overnight. Following resuspension to a stock concentration of 40 mM in buffer (20 mM Tris–Cl (pH 8.5), 50 mM NaCl), the lipids were freeze–thaw cycled using liquid N_2_ and an ∼50 °C water bath and then extruded through 100 nm nucleopore polycarbonate track-etched membranes (Whatman, Avanti extruder) at 35–40 °C (>10 °C higher than the lipid *T*_m_). As required, proteoliposomes were generated by mixing the required amount of unfolded OmpA by rapid dilution of denatured protein in 8 M urea to 1 M urea, and allowing the protein to fold into the membrane overnight at room temperature.

### LPR-matched proteoliposome generation

Using the fraction folded of each OmpA-variant into DLPC liposomes (Fig. 3c), the initial LPR was calculated such that final, folded OmpA LPR should be 320 : 1 (OmpA-WT 290 : 1, OmpA-Pos 160 : 1, OmpA-Neg 260 : 1, OmpA-Neut 240 : 1 mol mol^−1^) achieved by rapid dilution of the denatured protein in 8 M urea to 1 M urea, and allowing the protein to fold into the membrane overnight at room temperature. Following folding, unfolded OmpA (of which there were different amounts remaining in solution for different OmpA variants) and the exposed OmpA C-terminal domains were cleaved with 1 : 10 (mol mol^−1^) trypsin incubated for 3 h at 37 °C. Trypsin, peptides and any remaining unfolded protein were removed *via* two rounds of liposome pelleting *via* ultracentrifugation (110 000*g*, 4 °C, 30 min, Optima MAX-XP, Beckman Coulter). Prior to each centrifugation run, 20.1% (w/v) phenylmethylsulfonylchloride (PMSF) was added to inhibit residual trypsin. This method was validated by SDS-PAGE and Dynamic Light Scattering (DLS) (the latter using a Wyatt miniDAWN TREOS^®^ instrument). DMPC proteolipsomes (for lipid *T*_m_ measurements only) were made similarly, but during initial OMP folding were incubated at 24 °C overnight.

To estimate the number of OmpA molecules per liposome, the average proteoliposome hydrodynamic radius obtained by DLS (58 nm) was used. DLPC was assumed to occupy an area of 0.62 nm^2^ and to generate a bilayer thickness of 3 nm. There are thus ∼115 000 lipids per liposome. Given a final LPR of 320 : 1, there are on average ∼359 OmpA molecules per liposome, with a likely range of 300–400 for a typical-sized proteoliposome.

### Folding kinetics and urea titration

All folding kinetic measurements were performed using a BMG Clariostar platereader measuring intrinsic protein fluorescence (excitation 280 nm, emission 335 nm, both with 10 nm windows) at 10–30 s intervals, in sealed UV-transparent 96-well plates (CORNING 3635) at 30 °C in a 20 mM Tris–Cl (pH 8.5), 50 mM NaCl buffer (125 μL volumes). For folding reactions, proteoliposomes containing 1.25 μM of the pre-folded OmpA variant of interest were rapidly mixed with 1.25 μM unfolded OmpA (initial [urea] 8 M, final [urea] 1 M). Data were fitted to a single exponential or sigmoidal logarithmic curve to minimize the error, and the *T*_50_ values (time to reach 50% folded state) were extracted from the fit using Python. For display, up to a five-point moving average was applied to the data. For the urea titration, 2.5 μM of the pre-folded OmpA variant of interest in the proteoliposomes and 2.5 μM of unfolded OmpA were used. An initial 100-point reading was taken, before incubation overnight at 30 °C and a final 100-point measurement. The relative fraction of folded protein was determined by averaging the data over the final measurement for each individual condition and, if a plateau was reached at low urea concentrations, the data were normalised. Where possible, a sigmoidal logarithmic curve was fitted, and the urea concentration at the folding midpoint (*C*_m_) extracted. For the kinetics, significant differences were determined by permutation testing^58^ (which makes no assumption about the underlying distribution of the data), with the test statistic defined as the average difference between a pair of datasets. For the urea titration experiments, the significance was determined using paired *t*-tests over all the raw datapoints, with points paired for the same urea concentration and replicate.

### Laurdan measurement of lipid *T*_m_

Measurement of the lipid *T*_m_ in different proteoliposomes was performed using laurdan fluorescence as previously described.^11^ Briefly, DMSO-dissolved laurdan was added to pre-formed DMPC proteoliposomes at a lipid : laurdan ratio of 3200 : 1 (mol mol^−1^) (0.1% (v/v) DMSO final) and incubated overnight at room temperature. Fluorescence emission at 440 nm and 490 nm (excitation: 340 nm) was then measured at 0.5 °C intervals from 20–29 °C using a PTI fluorimeter (Horiba). General polarization (GP) was determined from the average intensity (*I*) at 440 and 490 nm, where GP = (*I*_440_ − *I*_490_)/(*I*_440_ + *I*_490_). Mid-points were determined by numerically differentiating the data.

### DLS

Proteoliposomes were diluted to a lipid concentration of ∼4 μM and 300 μL was injected into a Wyatt miniDAWN TREOS^®^ instrument. ∼5 min baselines were measured with filtered (0.22 μm) buffer (20 mM Tris–Cl (pH 8.5), 50 mM NaCl) before and after sample injection. The flow cell was flushed with 0.5 mL of 0.22-μm-filtered 1 M nitric acid and 1 mL 18 MΩ H_2_O after each run, followed by 1 mL of buffer. Correlation curves were analysed from a 3 min sample window by regularisation using Astra 6.0.3^®^.

### SDS-PAGE

Samples for SDS-PAGE analysis were mixed in a ratio of 1 : 3 with loading dye (50 mM Tris–HCl, pH 6.8, 6% (w/v) SDS, 0.3% (w/v) bromophenol blue, 40% (v/v) glycerol), boiled if required (>10 min, >95 °C) and ∼14 μL sample loaded into the gel (15% (w/v) Tris–tricine gels with 0.1% (w/v) SDS); ladder: Precision Plus Protein Dual Xtra Standards (BioRad). Following staining (InstantBlue Coomassie, Abcam), the gels were imaged using an Alliance Q9 imaging system (Uvitec) and densitometric analysis was performed using ImageJ. Where required, the folded fraction was calculated using the intensity ratio (folded/(folded + unfolded)) of the monomer bands.

## Results

### Electrostatic modelling of protein-induced charge asymmetry

To create controllable, protein-induced asymmetry in synthetic membranes, the insertion directionality and asymmetric property of the protein (here, the charge distribution), and the lipid : protein ratio must be known. Urea unfolded OmpA readily and quantitatively folds unidirectionally *in vitro* into pre-prepared liposomes of different lipid type (*e.g.* DMPC,^34^ DMPG,^11^ POPC^59^), oriented with its extracellular loops inside the liposome and its water-soluble C-terminal domain on the outside as it cannot cross the membrane.^11,34^ The extracellular loops of natively folded OmpA contain seven positively charged residues and seven negatively charged residues, while the intracellular turns contain four negative residues and two positive residues,^35^ generating a mild global dipole away from the more negative intracellular turns (Fig. 1). Additional charged residues exist within the core of the barrel, but these are secluded from the bulk solvent and engage in salt-bridges to stabilise the protein's core.^60^

**Fig. 2:**
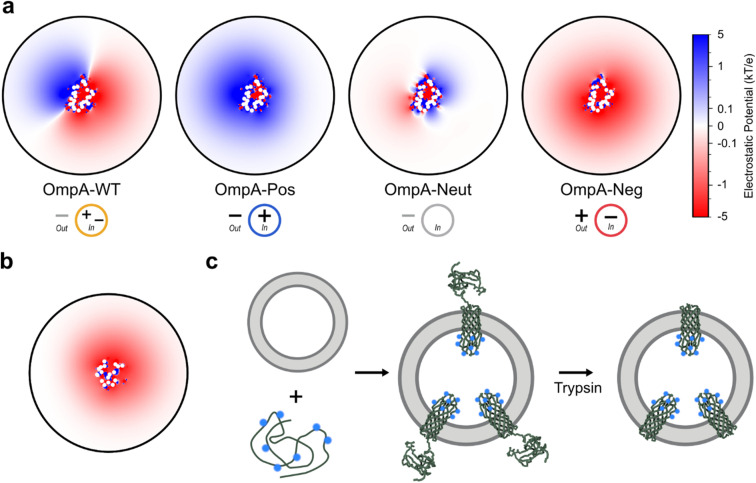
Modelling protein-induced charge-asymmetric liposomes. (a) Modelled electrostatic potential above the membrane plane around the extracellular loops of OmpA variants in DLPC membranes. The direction of the dipole generated from proteoliposomes of each variant is shown beneath each potential map. (b) Modelled electrostatic potential below the membrane plane around the intracellular turns of OmpA (in (a) and (b) white dots are in-view Cα; circular area is equivalent to the 320 : 1 (mol mol^−1^) experimental lipid : protein ratio used, ∼1 nm solvent slab analysed parallel to the membrane plane). (c) Experimental approach to generate protein-induced charge dipoles over the membrane: OmpA variants are unidirectionally inserted into pre-formed liposomes to a defined concentration, and then the soluble C-terminal domain is cleaved off using trypsin and the resultant proteoliposomes purified. The charge on the extracellular loops (here inside the liposomes when OmpA is folded) are altered by mutation (OmpA-Pos is shown here as an example, with blue positively charged symbols showing the seven positively charged residues in its extracellular loops).

Sequence variants of OmpA with altered charge in the extracellular loops were previously generated,^11^ notably OmpA-Pos (neutralisation of the seven negative residues, Fig. 1), OmpA-Neg (neutralisation of the seven positive residues, Fig. 1) and OmpA-Neut (neutralisation of all the positive and negative residues in the extracellular loops, Fig. 1). To better understand how these variants of OmpA manipulate the local electrostatic environment, the potential in a plane close to the membrane was modelled for each protein individually. A single copy of each OmpA variant was placed in a DLPC membrane with 50 mM NaCl (the same concentration used experimentally below), equilibrated, and then the electrostatic potential ∼0.8 nm above the membrane (*i.e.*, adjacent to the extracellular loops) was determined (Fig. 1 & 2a). The data show that the OmpA-Pos and OmpA-Neg variants generate a large area of electropositivity and electronegativity, respectively. The OmpA-Neut variant has a minimal charge footprint, while OmpA-WT has a split local potential with different sides of the protein being oppositely charged. The potential around the intracellular turns on the opposite side of the membrane was also assessed (Fig. 2b) which, as expected, showed a weak negative potential (average potential, excluding protein, over the area shown is −0.11 kT/e (intracellular turns); for comparison, OmpA-Neg is −0.48 kT/e at the extracellular loops). Together, this difference enables the dipole directionality to be assigned, as indicated below each potential profile in Fig. 2a.

### Generation and validation of charge-asymmetric proteoliposomes

Exploiting the different membrane dipoles induced by the OmpA variants enables proteoliposomes with membrane-protein-induced opposite charge dipoles to be created, with OmpA-Pos creating positive-inside proteoliposomes and OmpA-Neg negative-inside proteoliposomes, while OmpA-Neut has a mild positive-inside dipole. To create such protein-induced dipole asymmetric proteoliposomes, OmpA variants were folded into 100 nm diameter liposomes formed from DLPC to a known final LPR, and then the C-terminal domain was removed by cleavage with trypsin before purification using ultracentrifugation (see Methods) (Fig. 2c). DLPC was chosen for the experiments because of its net neutral charge (ensuring that the majority of the generated charge dipole arises from folded OmpA in the bilayer) and to ensure efficient folding of all the OmpA variants used (previous work showed maximum folding efficiencies of OMPs into short acyl chain lipids^38^).

**Fig. 3:**
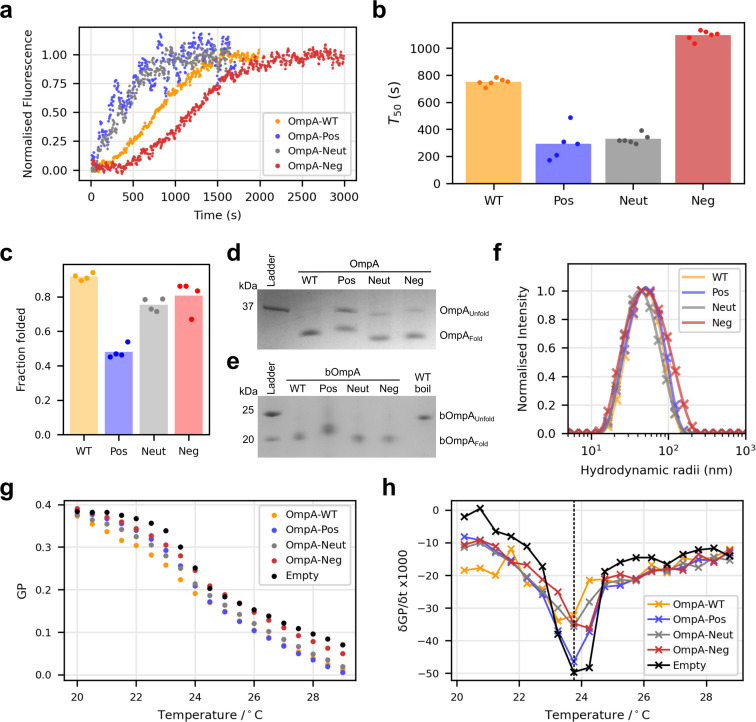
OmpA folding into DLPC and proteoliposome validation. (a) Example folding kinetics for the four OmpA variants inserting into empty DLPC liposomes and (b) fitted half-times (*T*_50_) for each curve (*n* ≥ 5). (c) Fraction of each OmpA variant folded into DLPC liposomes at an LPR of 320 : 1 (mol mol^−1^). (d) By matching the LPR to the yield of each folded protein, proteoliposomes with similar concentrations of each natively folded OmpA variant can be generated (original image in ESI Fig. 3†). (e) After cleavage of the C-terminal soluble domain with trypsin and purification, proteoliposomes with a similar LPR containing only the (folded) barrel domain of OmpA are recovered (original image in ESI Fig. 4†). (f) DLS of the final proteoliposomes used for folding assays. (g) The GP (generalised polarisation) ratio of laurdan fluorescence at 440 nm and 490 nm against temperature for DMPC liposomes containing each OmpA variant. (h) The first derivative of the GP indicates only small changes in the lipid *T*_m_ (curve minima) in the presence of each protein compared to empty liposomes (dashed line).

First the folding of all four OmpA variants into 100-nm extruded DLPC liposomes was measured at an LPR of 320 : 1 (mol mol^−1^) *via* intrinsic fluorescence (of OmpA's five tryptophans), creating proteoliposomes containing 300–400 OmpA molecules per liposome (see Methods). These experiments showed that folding for all proteins is completed by ∼2500 seconds (Fig. 3a). The time to reach half-maximum fluorescence, the *T*_50_, showed that OmpA-Pos and OmpA-Neut fold the fastest, with *T*_50_ values of ∼350 s, while OmpA-Neg folds slowest (*T*_50_ ∼1100 s) and OmpA-WT is intermediate (*T*_50_ ∼750 s) (Fig. 3b), highlighting the importance of the positive charge in the extracellular loops for efficient folding, consistent with previous results.^11^ They also show that the shorter-chain lipid (DLPC) enables a substantially faster (>3-fold) folding rate compared with folding of the same proteins into DMPC liposomes.^11^

Owing to the inherent high stability of its natively folded β-barrel, OmpA does not unfold in SDS detergent and migrates anomalously in cold SDS-PAGE, while non-native conformers of OmpA are SDS-sensitive^31^ (ESI Fig. 1†). This difference in electrophoretic mobility can be used to determine the yield of folded (native) protein *via* gel densitometry of samples analysed at the end of the folding reactions (Methods). Assessing these data for the different variants of OmpA analysed here (Fig. 3c) showed yields of natively folded protein ranging from ∼90% for OmpA-WT to ∼50% for OmpA-Pos. It is intriguing that the OmpA-Pos, while folding rapidly, results in a significantly lower folded yield than the other OmpA variants. This suggests that once ∼50% of the molecules have been folded into the bilayer, folding and membrane insertion of additional molecules is precluded. Given the known folding efficiencies, it is possible to conduct the folding reaction at LPRs tuned to each OmpA variant, such that the final amount of folded OmpA is approximately the same for each variant (with differing amounts remaining unfolded in solution) (ESI Fig. 2†). Thus, proteoliposomes containing approximately the same amount of folded protein content were generated (Fig. 3d). Upon the addition of trypsin to cleave the C-terminal domain of OmpA, any remaining unfolded protein is also digested and, following liposome purification by ultracentrifugation, only the folded OmpA barrels (bOmpA) at approximately equal protein concentration remain (Fig. 3e). Dynamic light scattering (DLS) confirmed that the proteoliposomes remain intact after this processing (Fig. 3f).

Although the barrel domains of OmpA are identical, it is possible that the differences in the extracellular loops of the OmpA variants may alter the membrane properties. To test for this, the global lipid phase transition temperature for the different OmpA proteins folded into DMPC liposomes (*T*_m_ 24 °C (without protein)) was assessed (the *T*_m_ of DLPC is −2 °C, making experiments with this lipid unfeasible). Accordingly, the OmpA variants were folded into 100-nm liposomes of DMPC at an LPR of ∼320 : 1 (mol mol^−1^). Lipid phase transition temperatures were then measured using the fluorescent probe laurdan, which changes its fluorescence profile depending on the lipid phase.^61^ The resulting sigmoidal curves with respect to temperature (Fig. 3g) were then differentiated to determine the *T*_m_ (Fig. 3h). Although slight differences (≤1 °C differences in *T*_m_) are apparent, the *T*_m_ of all proteoliposomes are within 0.5 °C of the empty liposomes, demonstrating minimal consequences of the presence of the different proteins on lipid order.

### Protein-induced dipoles modulate OmpA variant folding rates

**Fig. 4:**
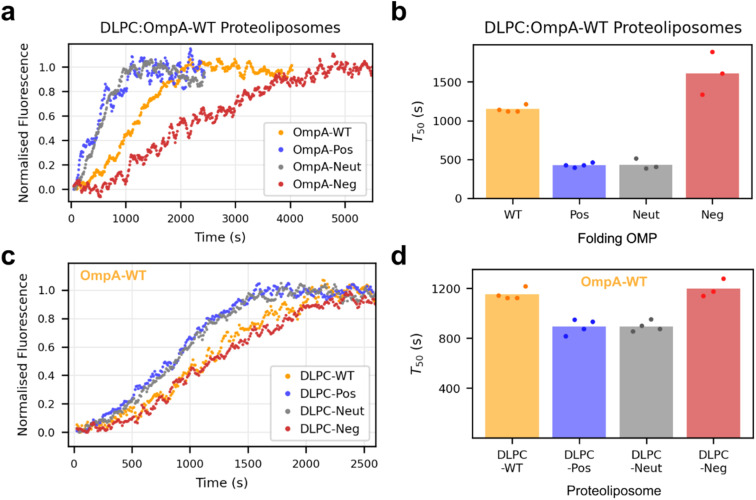
Both OmpA sequence and the charge dipole modulate the folding kinetics. (a) Sample kinetic traces of OmpA variants folding into DLPC:OmpA-WT proteoliposomes and (b) fitted *T*_50_ values for each curve (*n* ≥ 3). (c) Sample kinetic traces for OmpA-WT folding into proteoliposomes with different dipoles and (d) fitted *T*_50_ values for each curve (*n* ≥ 3). Data for DLPC-WT are reproduced from (b) for ease of comparison (the LPR of all proteoliposome substrates is ∼320 : 1).

To determine how the different protein-induced membrane charge-dipoles generated affect folding, full-length OmpA of each variant (OmpA-WT, OmpA-Pos, OmpA-Neut or OmpA-Neg (Fig. 1)) was folded into DLPC-proteoliposomes containing the pre-folded bOmpA variants as described above (named DLPC-WT/-Pos/-Neut/-Neg) (Fig. 2c). Similar to the empty DLPC liposomes, OmpA-Pos and OmpA-Neut fold more rapidly into DLPC-WT proteoliposomes than OmpA-WT, while the OmpA-Neg folds the slowest (Fig. 4a and b). Despite the high protein concentration in the membrane (final LPR of ∼160 : 1 (mol mol^−1^)), OmpA-Pos and OmpA-Neut fold at comparable rates into DLPC-WT as they do into the empty liposomes (*T*_50_ values of ∼350 and ∼400 s, respectively), while OmpA-WT folds about 50% more slowly (*T*_50_ values of ∼1150 and ∼750 s, respectively), as does OmpA-Neg (*T*_50_ values of ∼1610 and ∼1100 s, respectively) (Fig. 3b & 4b). These observations show that the charge in the extracellular loops of the folding OMP plays a role in determining the rate of folding (the folding rates follow the same rank order in DLPC and DLPC-WT), but the magnitude of the effect of the bilayer charge dipole depends on the charge in the extracellular loops of the folding OMP variant.

Next, OmpA-WT was folded into bilayers with different protein-induced dipoles (DLPC-WT/-Pos/-Neut/-Neg) (Fig. 4c). For OmpA-WT (that has charge-balanced positive and negative charges in its extracellular loops (Fig. 2a)), folding into positive-inside proteoliposomes (DLPC-Pos) was significantly (∼40%) faster than folding into negative-inside proteoliposomes (DLPC-Neg) (*T*_50_ values of ∼ 900 s and 1200 s, respectively) (Fig. 4d). Intriguingly, the folding rate enhancement observed here is opposite to that previously observed when studying the effects of lipid-induced dipoles,^11^ likely a result of differences in the two systems, including dipole magnitude (∼0.04 *vs.* ∼0.1–0.35 charge per lipid equivalent) and LPR (final LPR of 160 : 1 *vs.* 1600 : 1) for protein-induced *versus* lipid-induced membrane dipoles, respectively.^11^ Folding into DLPC-Neut occurs at the same rate as with DLPC-Pos (Fig. 4d). Together, these data highlight that there is an interplay between the charge on the protein and the local charge dipole across the bilayer that together modulate the rate of folding.

### Electrostatic matching between proteoliposomal dipole and folding OMP

**Fig. 5:**
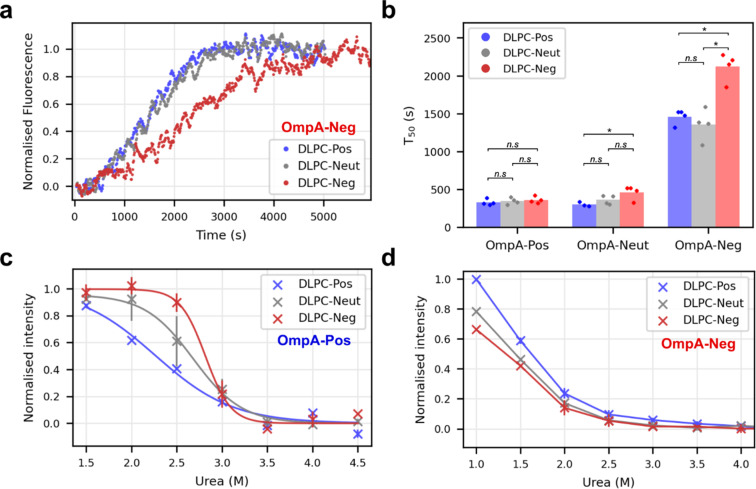
Electrostatic matching between folding OMP and proteoliposome dipole. (a) Sample kinetic traces of OmpA-Neg folding into differently dipoled proteoliposomes as indicated in the key. (b) Comparison of folding *T*_50_ values for OmpA-Pos, OmpA-Neut and OmpA-Neg into differently dipoled proteoliposomes. *P*-values were determined by permutation testing (*: *p* = 0.028, n.s.: not significant) (*n* ≥ 3). (c) Urea dependence of OmpA-Pos folding into differently dipoled proteoliposomes (*n* = 2, error bars show data range). Curves are fitted to the average data (bold symbols). (*P*-values: DLPC-Pos/DLPC-Neg: 0.014, DLPC-Pos/DLPC-Neut: 0.040, DLPC-Neg/DLPC-Neut: 0.135). (d) Urea dependence of OmpA-Neg folding into differently dipoled proteoliposomes (*n* = 2, error bars show data range, *n* = 1 for values at 1 M urea). Lines join the points and are to guide the eye only (*P*-values: DLPC-Pos/DLPC-Neg: 0.010, DLPC-Pos/DLPC-Neut: 0.010, DLPC-Neg/DLPC-Neut: 0.042) (LPR of the proteoliposome substrates in all panels is ∼320 : 1).

How the interaction between OmpA sequence charge and the protein-induced membrane dipole charge affects folding was considered next by comparing the folding rate of OmpA-Pos, OmpA-Neut and OmpA-Neg into proteoliposomes with different dipoles, *i.e.*, DLPC-Pos, DLPC-Neut and DLPC-Neg. The results showed that OmpA-Neg folds more slowly than all other variants tested into all types of proteoliposomes (Fig. 5a and b), with folding into DLPC-Neg being significantly slower than folding into DLPC-Neut and DLPC-Pos (Fig. 5b). In contrast, OmpA-Pos folds rapidly, and at a similar rate, into these three proteoliposome systems (Fig. 5b). Notably, however, this variant folds ∼6-fold more rapidly into DLPC-Neg (*T*_50_ of ∼350 s (Fig. 5b)) compared to OmpA-Neg (*T*_50_ of ∼2100 s (Fig. 5b)). Folding rates of OmpA-Neut are similar to OmpA-Pos, but with folding into DLPC-Neg slightly, but significantly, retarded relative to DLPC-Pos. Overall, therefore, the results show that the charge in the OmpA extracellular loops determines the folding rate, with a positive charge facilitating rapid folding. In addition, they reveal that the rate of folding is also dependent on the membrane charge dipole induced by pre-folding OmpA into the membrane, with the magnitude of the effect observed depending on a complex (and not yet understood) balance between the charge on the folding OmpA and the dipole induced across the bilayer by natively folded OmpA into the membrane.

Finally, the effect of urea on the yield of folded OmpA-Pos and OmpA-Neg was assessed by measuring the magnitude of the intrinsic fluorescence change at equilibrium at different urea concentrations when each protein was folded into the different proteoliposomes. Note that natively folded, membrane-embedded OmpA cannot be unfolded, even at high concentrations of urea, hence Δ*G*° values cannot be calculated.^31^ These experiments showed that OmpA-Pos is significantly more stable than OmpA-Neg in all bilayer types (Fig. 5c and d). For OmpA-Pos, the fractional folding curves plateaued at low urea concentrations allowing transition curves to be fitted and urea mid-point concentrations (*C*_m_) determined (Fig. 5c). OmpA-Pos folding into proteoliposomes with a complementary dipole (DLPC-Neg) had a *C*_m_ of 2.8 M urea, while folding the same protein into DLPC-Pos had a significantly lower *C*_m_ of 2.2 M urea (*p*-value = 0.015), while the *C*_m_ for DLPC-Neut lies in between these values (*C*_m_ of 2.6 M). OmpA-Neg is too unstable in all proteoliposomes to enable values of *C*_m_ to be determined, although the data suggest that the proteoliposomes with a complementary dipole (DLPC-Pos) support higher folding yields for a given urea concentration than those with non/less-complementary dipoles (DLPC-Neg/-Neut) (DLPC-Pos/DLPC-Neg *p*-value = 0.01) (Fig. 5d). Together the data are suggestive of a complementary electrostatic interaction between the folding OMP and protein-induced dipole over the membrane in determining the rate of folding and stability of OmpA in the bilayer.

## Discussion

The presence of proteins in biological membranes can confer a range of asymmetric properties on the bilayer, but the effects of these asymmetries on membrane-protein behaviour is largely unknown. Here we generated protein-induced transmembrane dipoles synthetically by exploiting modified forms of OmpA as a charge-carrying scaffold. OmpA forms an ideal platform for this purpose due to its ease of expression/purification, its ability to fold rapidly and efficiently *in vitro* into synthetic lipid bilayers of varied composition, and the high stability of its native state (∼−35 kJ mol^−1^ (ref. 62)). Furthermore, the ability to engineer its long loops without preventing folding^60,63,64^ and the presence of its 15 kDa C-terminal soluble domain that is unable to cross the membrane and enforces unidirectional folding^34^ provide the attributes required to build proteoliposomes with different protein-induced dipoles across the membrane. While the effects of charge dipoles on folding were explored here, the properties of OmpA make it a broadly applicable scaffold to explore other types of protein-induced membrane asymmetries. For example, by engineering the protein sequence further, the consequences of asymmetric molecular crowding, differential amino acid properties in each bilayer leaflet or the introduction of leaflet-specific lipid binding sites could be explored.

We have shown here that manipulating the protein-induced electrostatic dipole across a lipid bilayer alters the folding rate of OmpA in a manner that is dependent both on the charge in the extracellular loops of the folding protein and the protein-induced dipole across the membrane. Most strikingly, it was found that proteoliposomes support faster folding and more effective stabilisation when the charge dipole of the membrane is complementary to that of the folding protein, as exemplified by OmpA-Neg folding into DLPC-Pos. However, the relationship is complex, since OmpA-Pos folds with a similar rate into DLPC-Pos/-Neg/-Neut. This could reflect the rapid intrinsic folding rate of OmpA-Pos into all three bilayers, making it difficult to detect kinetic differences, or reflect a change in folding mechanism in which charge effects are not rate determining. In addition, interaction of the charge on the folding protein with the short turns on bOmpA-loaded proteoliposomes, which are net negative (Fig. 2a) and exposed on the surface of the liposome, may also influence the rates of folding, possibly by electrostatically disfavouring the approach of OmpA-Neg to the membrane, while facilitating binding, and hence folding, of OmpA-Pos. It should also be borne in mind that the lipid charge and lipid-induced dipole across the membrane can also affect the folding rate, as shown previously,^11^ making it difficult to generate ‘rules’ that rationalise the effects for the set of protein charges and membrane dipoles examined here. Further experiments using different OMPs, membranes of different lipid compositions, and with different protein- and lipid-based asymmetries will be needed to create a database of sufficient size to generate such rules.

Intriguingly, while we show here that OmpA-WT folds 40% more rapidly into positive-inside proteoliposomes compared to negative-inside proteoliposomes (Fig. 4d), we previously reported that the same protein folds up to ∼10-times more slowly into liposomes with a lipid-induced positive-inside charge dipole, *i.e.*, a strong effect in the opposite direction was observed.^11^ Although the exact nature of this difference remains unclear, it could arise from the different LPRs used in the different experiments (final LPR of 160 : 1 *vs.* 1600 : 1), with the high concentrations of protein in the membranes used in this study altering the mechanism of the folding process. It could also result from the relatively small dipoles created in this study compared to those generated by asymmetric lipid organisation (∼0.04 *vs.* ∼0.1–0.35 charge per lipid equivalent). This again highlights the complexity of the pathways of membrane protein folding in these ‘simple’ synthetic membrane systems, and raises the intriguing question of how such effects may manifest in the more complex situation of the bacterial OM.

In summary, the results presented here demonstrate that protein-induced membrane-dipole asymmetries can modulate OmpA folding rates and stability. Specifically, protein-induced bilayer dipoles are shown to be able to change the folding rates of OmpA by up to a 6-fold increase in a manner that depends both on the charge complementarity between the folding protein and the membrane that together contribute to defining the folding rate. Our results suggest new approaches that could be used to enhance the creation and stabilisation of OMPs in bilayers for use in biotechnology.^49–51^ Equally importantly, they also show how the crowded and highly asymmetric bacterial OM might profoundly modulate the folding and properties of the embedded OMPs. They also highlight how bacteria may alter their proteomes to stabilise and/or accelerate (and *vice versa*) the folding/localisation of specific proteins to allow for concerted membrane adaptation. Given the low LPR in the protein-rich OM in which OMPs are highly crowded, and in which OmpA is one of the most abundant OM proteins,^32^ it seems likely that at least some of these consequences will be important *in vivo*.

## Author contributions

All authors designed the experiments. J. M. M. performed all the research. All authors contributed to the analysis of the data, and all authors wrote or edited the manuscript.

## Conflicts of interest

The authors have no conflict of interest.

## Supplementary Material

FD-259-D4FD00180J-s001

## Data Availability

Source data files containing fluorescence folding traces, gel images, DLS and electrostatic potential files are freely available at the University of Leeds Data Repository (https://doi.org/10.5518/1603).

## References

[cit1] Harayama T., Riezman H. (2018). Understanding the diversity of membrane lipid composition. Nat. Rev. Mol. Cell Biol..

[cit2] Cheng X., Smith J. C. (2019). Biological membrane organization and cellular signaling. Chem. Rev..

[cit3] Nagata S., Suzuki J., Segawa K., Fujii T. (2016). Exposure of phosphatidylserine on the cell surface. Cell Death Differ..

[cit4] Bogdanov M. (2020). *et al.*, Phospholipid distribution in the cytoplasmic membrane of Gram-negative bacteria is highly asymmetric, dynamic, and cell shape-dependent. Sci. Adv..

[cit5] Maures T. J., Su H.-W., Argetsinger L. S., Grinstein S., Carter-Su C. (2011). Phosphorylation controls a dual-function polybasic nuclear localization sequence in the adapter protein SH2B1β to regulate its cellular function and distribution. J. Cell Sci..

[cit6] Beer K. B. (2018). *et al.*, Extracellular vesicle budding is inhibited by redundant regulators of TAT-5 flippase localization and phospholipid asymmetry. Proc. Natl. Acad. Sci. U. S. A..

[cit7] Jia W. (2004). *et al.*, Lipid trafficking controls endotoxin acylation in outer membranes of *Escherichia coli*. J. Biol. Chem..

[cit8] Rothman J. E., Lenard J. (1977). Membrane asymmetry. Science.

[cit9] van Meer G. (2005). Cellular lipidomics. EMBO J..

[cit10] Pabst G., Keller S. (2024). Exploring membrane asymmetry and its effects on membrane proteins. Trends Biochem. Sci..

[cit11] Machin J. M., Kalli A. C., Ranson N. A., Radford S. E. (2023). Protein–lipid charge interactions control the folding of outer membrane proteins into asymmetric membranes. Nat. Chem..

[cit12] Lin Q., London E. (2014). The influence of natural lipid asymmetry upon the conformation of a membrane-inserted protein (perfringolysin O). J. Biol. Chem..

[cit13] Scott H. L., Heberle F. A., Katsaras J., Barrera F. N. (2019). Phosphatidylserine asymmetry promotes the membrane insertion of a transmembrane helix. Biophys. J..

[cit14] Sharpe H. J., Stevens T. J., Munro S. (2010). A comprehensive comparison of transmembrane domains reveals organelle-specific properties. Cell.

[cit15] Drew D., Boudker O. (2024). Ion and lipid orchestration of secondary active transport. Nature.

[cit16] Lorent J. H. (2020). *et al.*, Plasma membranes are asymmetric in lipid unsaturation, packing and protein shape. Nat. Chem. Biol..

[cit17] Buyan A. (2020). *et al.*, Piezo1 Forms Specific, Functionally important interactions with phosphoinositides and cholesterol. Biophys. J..

[cit18] Löwe M. (2023). *et al.*, Probing macromolecular crowding at the lipid membrane interface with genetically-encoded sensors. Protein Sci..

[cit19] Yang X. (2022). *et al.*, Structure deformation and curvature sensing of PIEZO1 in lipid membranes. Nature.

[cit20] Banerjee T. (2022). *et al.*, Spatiotemporal dynamics of membrane surface charge regulates cell polarity and migration. Nat. Cell Biol..

[cit21] Peruzzi J. A. (2024). *et al.*, Hydrophobic mismatch drives self-organization of designer proteins into synthetic membranes. Nat. Commun..

[cit22] Shelby S. A., Castello-Serrano I., Wisser K. C., Levental I., Veatch S. L. (2023). Membrane phase separation drives responsive assembly of receptor signaling domains. Nat. Chem. Biol..

[cit23] Li R., Zhao R., Yang M., Zhang X., Lin J. (2023). Membrane microdomains: Structural and signaling platforms for establishing membrane polarity. Plant Physiol..

[cit24] Jiang W. (2021). *et al.*,Crowding-induced
opening of the mechanosensitive Piezo1 channel in silico. Commun. Biol..

[cit25] Lin Y., Buyan A., Corry B. (2022). Computational studies of Piezo1 yield insights into key lipid–protein interactions, channel activation, and agonist binding. Biophys. Rev..

[cit26] Lessen H. J., Fleming P. J., Fleming K. G., Sodt A. J. (2018). Building blocks of the outer membrane: Calculating a general elastic energy model for β-barrel membrane proteins. J. Chem. Theory Comput..

[cit27] Rassam P. (2015). *et al.*, Supramolecular assemblies underpin turnover of outer membrane proteins in bacteria. Nature.

[cit28] Horne J. E., Brockwell D. J., Radford S. E. (2020). Role of the lipid bilayer in outer membrane protein folding in Gram-negative bacteria. J. Biol. Chem..

[cit29] Tan W. B., Chng S.-S. (2024). How bacteria establish and maintain outer membrane lipid asymmetry. Annu. Rev. Microbiol..

[cit30] Schulz G. E. (2002). The structure of bacterial outer membrane proteins. Biochim. Biophys. Acta, Biomembr..

[cit31] Schüßler A. , Herwig S. and Kleinschmidt J. H., Kinetics of Insertion and Folding of Outer Membrane Proteins by Gel Electrophoresis, in Lipid-Protein Interactions: Methods and Protocols, ed. J. H. Kleinschmidt, Methods in Molecular Biology, Humana, New York, NY, 2019, vol. 2003, pp. 145–162 10.1007/978-1-4939-9512-7_7 31218617

[cit32] Li G.-W., Burkhardt D., Gross C., Weissman J. S. (2014). Quantifying absolute protein synthesis rates reveals principles underlying allocation of cellular resources. Cell.

[cit33] Rojas E. R. (2018). *et al.*, The outer membrane is an essential load-bearing element in Gram-negative bacteria. Nature.

[cit34] Surrey T., Jähnig F. (1992). Refolding and oriented insertion of a membrane protein into a lipid bilayer. Proc. Natl. Acad. Sci. U. S. A..

[cit35] Pautsch A., Schulz G. E. (2000). High-resolution structure of the OmpA membrane domain. J. Mol. Biol..

[cit36] Andersen K. K., Wang H., Otzen D. E. (2012). A kinetic analysis of the folding and unfolding of OmpA in urea and guanidinium chloride: single and parallel pathways. Biochemistry.

[cit37] Bulieris P. V., Behrens S., Holst O., Kleinschmidt J. H. (2003). Folding and insertion of the outer membrane protein OmpA is assisted by the chaperone Skp and by lipopolysaccharide. J. Biol. Chem..

[cit38] Schiffrin B. (2017). *et al.*, Effects of periplasmic chaperones and membrane thickness on BamA-catalyzed outer-membrane protein folding. J. Mol. Biol..

[cit39] Gessmann D. (2014). *et al.*, Outer membrane β-barrel protein folding is physically controlled by periplasmic lipid head groups and BamA. Proc. Natl. Acad. Sci. U. S. A..

[cit40] Burgess N. K., Dao T. P., Stanley A. M., Fleming K. G. (2008). Beta-barrel proteins that reside in the *Escherichia coli* outer membrane *in vivo* demonstrate varied folding behavior *in vitro*. J. Biol. Chem..

[cit41] Danoff E. J., Fleming K. G. (2015). Membrane defects accelerate outer membrane β-barrel protein folding. Biochemistry.

[cit42] Zhang X. C., Li H. (2019). Interplay between the electrostatic membrane potential and conformational changes in membrane proteins. Protein Sci..

[cit43] Levental I., Lyman E. (2023). Regulation of membrane protein structure and function by their lipid nano-environment. Nat. Rev. Mol. Cell Biol..

[cit44] Clarke R. J. (2023). Electrostatic switch mechanisms of membrane protein trafficking and regulation. Biophys. Rev..

[cit45] von Heijne G. (1989). Control of topology and mode of assembly of a polytopic membrane protein by positively charged residues. Nature.

[cit46] Baker J. A., Wong W.-C., Eisenhaber B., Warwicker J., Eisenhaber F. (2017). Charged
residues next to transmembrane regions revisited: “Positive-inside rule” is complemented by the “negative inside depletion/outside enrichment rule”. BMC Biol..

[cit47] Jackups R., Liang J. (2005). Interstrand pairing patterns in beta-barrel membrane proteins: the positive-outside rule, aromatic rescue, and strand registration prediction. J. Mol. Biol..

[cit48] Slusky J. S. G., Dunbrack R. L. (2013). Charge asymmetry in the proteins of the outer membrane. Bioinformatics.

[cit49] An L. (2024). *et al.*, Binding and sensing diverse small molecules using shape-complementary pseudocycles. Science.

[cit50] Berhanu S. (2024). *et al.*, Sculpting conducting nanopore size and shape through de novo protein design. Science.

[cit51] Dorey A., Howorka S. (2024). Nanopore DNA sequencing technologies and their applications towards single-molecule proteomics. Nat. Chem..

[cit52] Arora A., Abildgaard F., Bushweller J. H., Tamm L. K. (2001). Structure of outer membrane protein A transmembrane domain by NMR spectroscopy. Nat. Struct. Biol..

[cit53] Ishida H., Garcia-Herrero A., Vogel H. J. (2014). The periplasmic domain of *Escherichia coli* outer membrane protein A can undergo a localized temperature dependent structural transition. Biochim. Biophys. Acta.

[cit54] Jo S., Kim T., Iyer V. G., Im W. (2008). CHARMM-GUI: A web-based graphical user interface for CHARMM. J. Comput. Chem..

[cit55] Lee J. (2016). *et al.*, CHARMM-GUI Input Generator for NAMD, GROMACS, AMBER, OpenMM, and CHARMM/OpenMM Simulations Using the CHARMM36 Additive Force Field. J. Chem. Theory Comput..

[cit56] Van Der Spoel D. (2005). *et al.*, GROMACS: fast, flexible, and free. J. Comput. Chem..

[cit57] Jurrus E. (2018). *et al.*, Improvements to the APBS biomolecular solvation software suite. Protein Sci..

[cit58] Box G. E. P., Andersen S. L. (1955). Permutation theory in the derivation of robust criteria and the study of departures from assumption. J. R. Stat. Soc. Ser. B Methodol..

[cit59] Hussain S., Bernstein H. D. (2018). The Bam complex catalyzes efficient insertion of bacterial outer membrane proteins into membrane vesicles of variable lipid composition. J. Biol. Chem..

[cit60] Smith S. G. J., Mahon V., Lambert M. A., Fagan R. P. (2007). A molecular Swiss army knife: OmpA structure, function and expression. FEMS Microbiol. Lett..

[cit61] White P. (2021). *et al.*, The role of membrane destabilisation and protein dynamics in BAM catalysed OMP folding. Nat. Commun..

[cit62] Lomize A. L., Todd S. C., Pogozheva I. D. (2022). Spatial arrangement of proteins in planar and curved membranes by PPM 3.0. Protein Sci..

[cit63] Franklin M. W. , Stevens J. J., Krise J. and Slusky J. S. G., The Extracellular Loops of OmpA Control the Slow Rate of *In Vitro* Folding, bioRxiv, 2021, preprint, 10.1101/2020.10.08.331546

[cit64] Koebnik R. (1999). Structural and functional roles of the surface-exposed loops of the beta-barrel membrane protein OmpA from *Escherichia coli*. J. Bacteriol..

