# Supplementary material for: Protein-induced membrane asymmetry modulates OMP folding kinetics and stability
Source: Faraday Discuss. 2025 Jan 10;259:579–96. doi: 10.1039/d4fd00180j (PMC12060775; doi:10.1039/d4fd00180j)
Supplement: FD-259-D4FD00180J-s001 [file FD-259-D4FD00180J-s001.pdf]

Supplementary Information (SI) for Faraday Discussions.
This journal is © The Royal Society of Chemistry 2025

Supplementary Figures

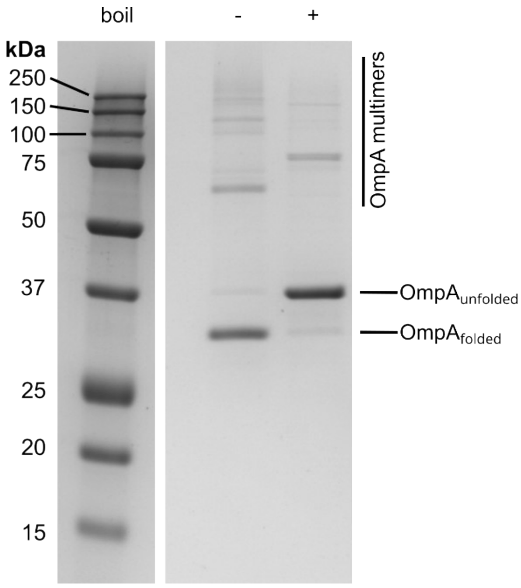

Supplementary Figure 1: OmpA bandshifts in cold SDS-PAGE depending on its folding state. The transmembrane domain of OmpA forms an SDS resistant fold in the absence of boiling, which causes a bandshift of folded (unboiled) compared to unfolded (boiled) OmpA, with the folded form running with a lower apparent molecular weight. Minor subpopulations of OmpA can self-associate to form a range of oligomeric species, even in the unfolded state.

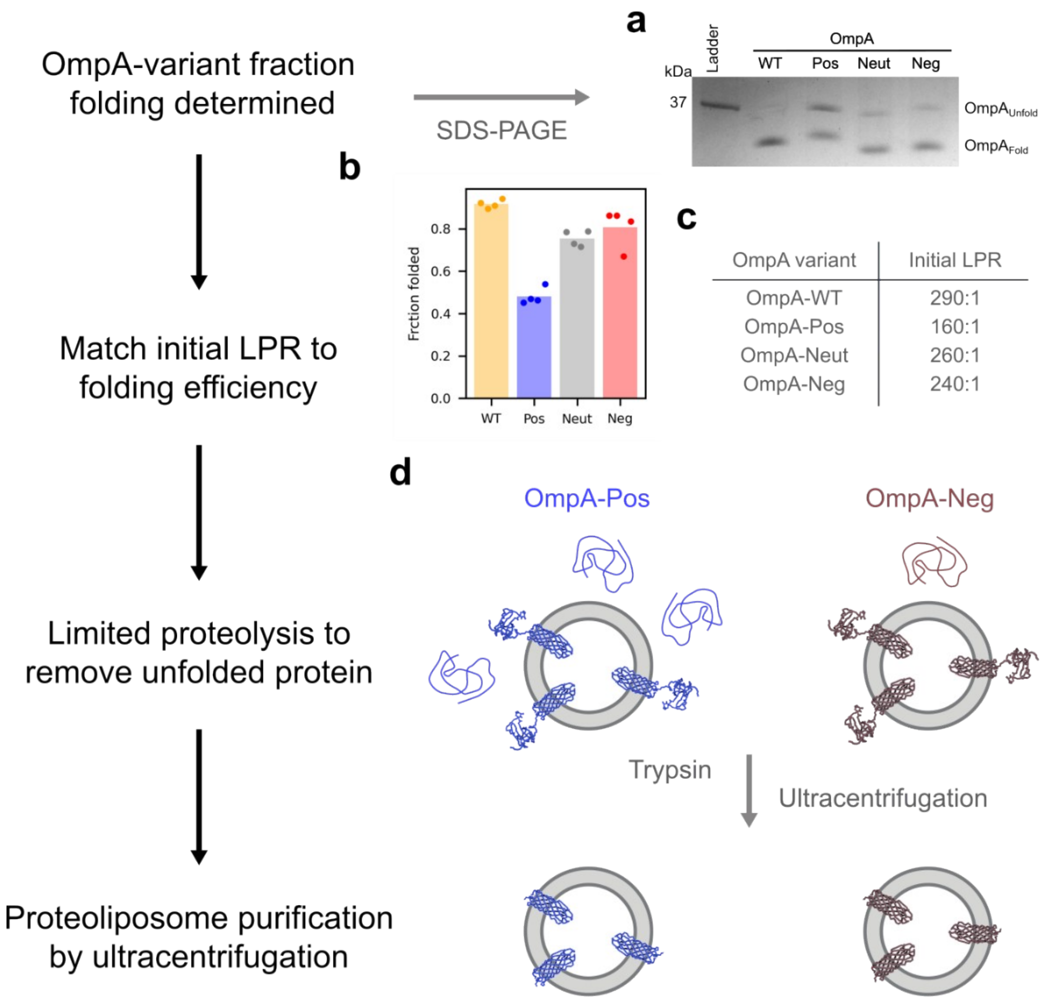

Supplementary Figure 2: Method to generate LPR matched proteoliposomes with OmpA charge variants. Initially the folding efficiency of each OmpA variant was assessed by cold SDS-PAGE (**a**), which enabled the fraction folded, membrane inserted protein to be determined (**b**). Guided by the fraction folded, the initial LPR was calculated such that the final, folded OmpA LPR should be 320:1 (**c**). Following folding, different amounts of unfolded protein remained in solution (**d**), which were then cleaved, along with OmpA's C-terminal domain, using trypsin. The LPR matched proteoliposomes were then purified via ultracentrifugation and validated (see **Figure 3**).

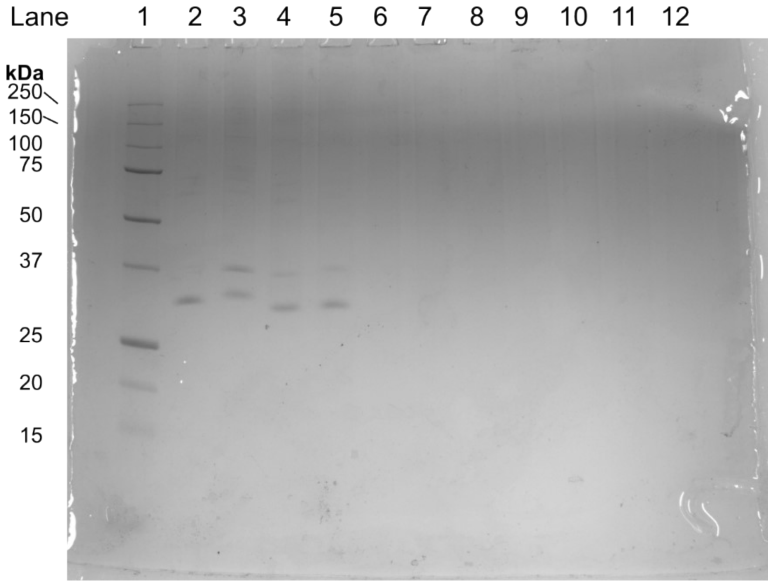

Supplementary Figure 3: Original SDS PAGE gel image for Figure 3d. Lane 1: ladder, 2: DLPC + OmpA-WT, 3: DLPC + OmpA-Pos, 4: DLPC + OmpA-Neut, 5: DLPC + OmpA-Neg, 6-12: empty.

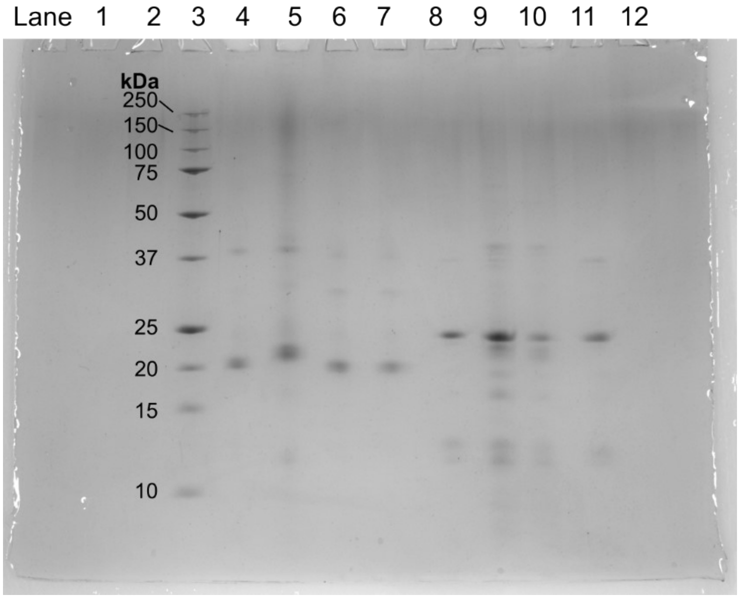

Supplementary Figure 4: Original SDS PAGE gel image for Figure 3e. Lane 1-2: empty, 3: ladder, 4: DLPC + bOmpA-WT, 5: DLPC + bOmpA-Pos, 6: DLPC + bOmpA-Neut, 7: DLPC + bOmpA-Neg, 8: DLPC + bOmpA-WT boiled, 9: DLPC + bOmpA-Pos boiled, 10: DLPC + bOmpA-Neut boiled, 11: DLPC + bOmpA-Neg boiled, 12: empty. The lower molecular weight bands in the lanes of boiled samples indicate some of the bOmpA is also cleaved, however, as the same sample migrates as a single-band in the unboiled lanes, a single intact barrel structure must be retained, thus the generated dipoles should not be affected by this population.
